# Pathogenic bacteria in cheese, raw and pasteurised milk

**DOI:** 10.1002/vms3.604

**Published:** 2021-08-14

**Authors:** Mahsa Morovati Bastam, Mahsa Jalili, Iraj Pakzad, Abbas Maleki, Sobhan Ghafourian

**Affiliations:** ^1^ Department of Microbiology Faculty of Medicine, Ilam University of Medical Sciences Ilam Iran; ^2^ Clinical Microbiology Research Center Ilam University of medical Sciences Ilam Iran

**Keywords:** identification, microbial contamination, milk

## Abstract

**Background:**

Foodborne diseases, especially those transmitted by milk and its products, are worldwide problem. Milk is not only a complete food but also a unique medium for activating various bacteria such as *Listeria monocytogenes*, *Staphylococcus aureus* and *Salmonella typhi*. In recent years, numerous bacteria with multiple drug resistance patterns have appeared, and there have been many problems in infection control. Today, ranchers use antibiotics for control of the animal disease, and humans are constantly using animal products containing antibiotics.

**Objective:**

The purpose of this study was to evaluate the contamination status of raw and pasteurised milk as well as local cheese and to find a rapid Multiplex PCR method for investigation of contamination. Determination of antibiotic resistant isolates is also desirable.

**Materials and Methods:**

One hundred samples were collected from livestock and retail outlets using culture and molecular methods to identify *S. aureus*, *L. monocytogenes* and *S. typhi*. The antibiotic resistance pattern was determined for the isolates.

**Results:**

In this study, culture results for 100 samples showed 10% *S. aureus* isolates while no cases of *S. typhi* and *L. monocytogenes* were detected. In real‐time qPCR, *S. aureus* was isolated in 60% (*n* = 60) of samples, *S. typhi* in 53% (*n* = 53) and *L. monocytogenes* in 2% (*n* = 2). The results of sensitivity and specificity of Multiplex PCR for the three studied bacteria indicated general specificity of 72% and sensitivity of 80%.

**Conclusion:**

Based on the results of this study, it can be concluded that *S. typhi*, *L. monocytogenes* and *S. aureus* are more likely to be detected by real‐time qPCR because of the high sensitivity of this test to culture. Multiplex method was not reliable in this study and cannot be suggested for rapid diagnosis.

## Graphical abstract

Because of consumption of some milk and dairy product as traditional in Ilam, Iran, we decided to evaluate the microbial contamination in raw milk, cheese and pasteurised milk by culture and molecular methods. Our results showed some of raw milk, cheese and pasteurised milk had microbial contamination.

We found the molecular methods are reliable to detect *S. aureus*, *S. typhi* and L*. monocytogenes*.

Although, Multiplex PCR is fast, but we could not suggest this method to detect the three studied bacteria.

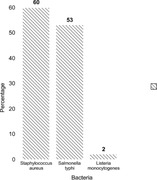

## 1 INTRODUCTION

Milk and dairy products have a special place among various foods of animal and plant origin. Adequate protein, fat, essential amino acids, and minerals like calcium and phosphorus reveal the importance of milk and dairy products (Kaskous & Pfaffl, 2017). In addition, millions of people consume milk and dairy products in their meals every day. Therefore, providing high quality products will be essential to ensure the health of the community. Given the importance of milk in human nutrition and general community health, knowledge of its components and the soundness of this important food product is necessary and inevitable (Singhal et al., 2017). Milk and its products contain different nutrients and the pH of milk is equal to 6.8; therefore, it is a good environment for the growth of many microorganisms. Milk has a primary microbial flora when milking cows, but it may be contaminated with other pathogenic microorganisms (Munera‐Bedoya et al., 2017). A number of diseases can be transmitted to humans through milk. Microbes that can be transmitted to milk through cattle lead to infection with *Staphylococcus aureus*, bovine tuberculosis, brucellosis, malaria, streptococcal and salmonellosis infections. Also, some contaminants may be transmitted through milk carriers. Transmission through contaminated soil and equipment can also lead to listeriosis and salmonellosis, as well as Campylobacter‐related infections in consumers (Nornberg et al., 2010). The health of raw milk is in the interest of public, and failure provide high quality milk will reduce the level of public health as well as economic health; therefore, due to the importance of this issue, it is vital to quickly and accurately identify raw milk contamination (Abushelaibi et al., 2017). Therefore, it is necessary to study the profile of pathogenic microbes phenotypically. Considering the importance of milk as an essential nutrient, it is advisable for the research community to identify pathogenic bacteria such as *Salmonella spp*, *S. aureus* and *Listeria monocytogenes* and to study the spread of antibiotic resistance in these pathogens (Marin et al., 2017). Therefore, the current study aimed to evaluate the contamination of raw and pasteurised milk as well as cheese with *Salmonella typhi*, *L.monocytogenes* and *S.aureus* to determine the antibiotic resistance pattern of the isolated bacteria.

## 2 MATERIALS AND METHODS

### 2.1 Sample collection

One hundred samples, including 35 samples of raw and 35 samples of pasteurised milk, as well as 30 samples of cheese were collected from diary and livestock centres.

### 2.1 Culture and identification of *S. aureus, S. typhi* and *L. monocytogenes*

In each plate, after pouring milk or cheese homogenisation, culture was performed on MacConkey agar, blood agar and chocolate agar and incubated for 24 h at 37°C. After 24 h, the colonies suspected of being *S. aureus, S. typhi* and *L. monocytogenes* were examined. Initially, single colonies were expanded, gram stained and catalase tested. Then, all slides were studied under a light microscope. In the final stage, tubular coagulase and DNase tests was performed for *S. aureus* isolates. Motility tests were performed at 37°C and room temperature for isolates suspected of being *L. monocytogenes*. For *S. typhi*, after culture on Mac Conkey agar (Merck) and xylose lysine deoxycholate agar (XLD) (Merck) the specific tests of Enterobacteriaceae family, including catalase test, oxidase test, methyl red (MR) (Merck), Voges–Proskauer (VP) (Merck),citrate utilisation (Merck), sulfide indole motility (SIM) (Merck), Kligler Iron Agar (KIA) (Sigma‐Aldrich) and Lysine iron agar (LIA) (Merck) were performed, the suspected isolates were subsequently evaluated with specific anti‐sera (Bharafshan, Iran).

### 2.2 Antibiotic susceptibility test

To determine the antibiotic resistance patterns of common milk bacteria, antibiotic susceptibility assay was performed by disk diffusion. The antibiotics included ampicillin (10 µg), tetracycline (30 µg), gentamicin (10 µg), ceftazidime (30 µg), ciprofloxacin (5 µg), imipenem (10 µg), penicillin (10 µg), cotrimoxazole (25 µg), ceftriaxone (30 µg) and cefoxitin (30 µg). It should be noted that antimicrobial assay was performed only for *S. aureus* because only this bacterium was isolated from samples. Disks were purchased from the Padtan Teb Co. (Iran)

### 2.3 DNA extraction

DNA extraction was performed by boiling method (Oliveira et al., 2014).

### 2.4 Real‐time qPCR

Real‐time qPCR was performed to assess the presence of specific bacterial genes. For this purpose, special microtubes with 0.2 transparencies were used. After counting the microtubes, in each microtube, 12 µl of SYBR Green (Real‐Time PCR Master Mix) containing DNA polymerase, dNTP buffer system and SYBR Green fluorescent dye, 8 µl of nuclease free water, 1 µl of forward primer (final concentration of 5 picomoles/µl), 1 µl of reverse primer (final concentration of 5 picomoles/µl) and 3 µl of DNA (concentration of 100 ng/µl) were added to a final volume of 25 µl. Negative control method was used to investigate possible contamination. The microtubes were then placed in a real‐time machine (BioRAD, USA) and the necessary adjustments were made.

### 2.5 Multiplex PCR

For Multiplex PCR, the primers listed in Table 1 were used. The method applied to test may be suggested to accelerate the test.

**TABLE 1:** Primers used in Multiplex PCR

Gene	Sequence (5′→3′)	Sizes (bp)
*nuc*	F: ACAGAGGTAAACGCAACGA	126
	R: ACCTGTAACCGCACCAAGTT	
*hly A*	F:CAGGAATGACTAATCAAGACA	315
	R:AGGTTCATTAACAATCACG	
*Inv A*	F:CTTTGATAAACTTCATCGCAC	200
	TCGTTATTACCAAAGGTTCAG	

### 2.6 Determination of specificity and sensitivity of Multiplex PCR

Sensitivity and specificity are two important indicators for statistically evaluating the performance of binary classification test results, which are known in statistics as classification functions. When data can be divided into positive and negative groups, the performance of the results of an experiment that divides information into these two categories can be measured and described using sensitivity and specificity indexes.

**Sensitivity**: The proportion of positive items that the test correctly marks as positive.

$$\mathrm{Sensitivity}=\frac{\text{True}\;\text{positive}}{\text{False}\;\text{negative}+\text{true}\;\text{positive}}$$

Specificity: The proportion of negatives that the test correctly marks as negative.

$$\mathrm{Specificity}=\frac{\text{True}\;\text{negative}}{\text{True}\;\text{negative}+\text{false}\;\text{positive}}$$

## 3 RESULTS AND DISCUSSION

Our results showed that only *S. aureus* was isolated with frequency of 10% (*n* = 10) by culture method, which was detected in three samples out of a total of 30 cheese samples (11%) as well as seven samples from a total of 35 raw milk samples (22.8%). Also, our results showed two cheeses samples (5.7%) that were positive for *L. monocytogenes*.

### 3.1 Real‐time qPCR test to identify *S. aureus*, *S. typhi* and *L. monocytogenes* via *nuc*, *invA* and *hlyA* genes, respectively

**FIGURE 1:**
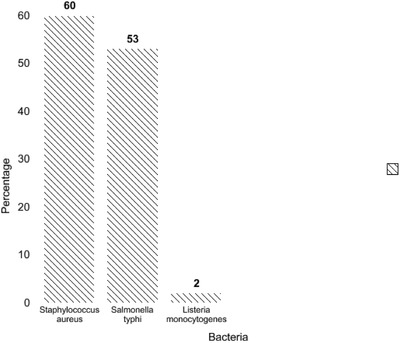
Identification of *S. aureus, S. typhi* and *L. monocytogenes* via *nuc, invA* and *hlyA* genes, respectively, by real‐time qPCR

Among 100 samples, 60% (*n* = 60) were positive for *S. aureus* via detection of *nuc* gene, 53% (*n* = 53) of samples were positive for *S. typhi* by investigation of *invA* gene and 2% (*n* = 2) were positive for *L.monocytogenes* through identification of *hlyA* gene (Figure 1 and Table 2).

**TABLE 2:** Identification of *S. aureus*, *S. typhi* and *L. monocytogenes* in cheese, raw and pasteurised milk by qPCR

Samples	Total number	Number of positive isolates	Percentage of positive isolates
*S. aureus*			
Raw milk	35	13	37.1%
Pasteurised milk	35	26	74.2%
Cheese	30	21	70%
Total	100	60	60%
*L. monocytogenes*			
Raw milk	35	2	5.7%
Pasteurised milk	35	0	0%
Cheese	30	0	0%
Total	100	2	2%
*S. typhi*			
Raw milk	35	18	51.4%
Pasteurised milk	35	20	57.1%
Cheese	30	15	50%
Total	100	53	53%

### 3.2 Multiplex PCR for identification of *S. aureus*, *S. typhi* and *L. monocytogenes* via *nuc*, *invA* and *hlyA* genes, respectively

**FIGURE 2:**
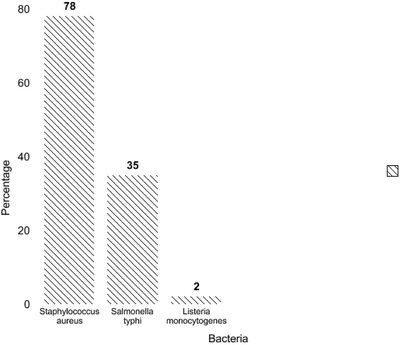
Identification of *S. aureus, S. typhi* and *L. monocytogenes* in cheese, raw and pasteurised milk by Multiplex PCR

**FIGURE 3:**
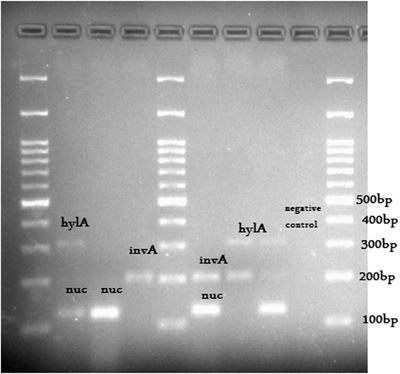
Multiplex PCR for nuc, invA and hylA genes in cheese, raw and pasteurised milk; nuc = 126bp, hylA = 315bp, invA = 200bp

The presence of *nuc, invA* and *hlyA* genes was evaluated in 100 samples of raw milk, pasteurised milk and local cheese with frequency of 35% (*n* = 35), 78% (*n* = 78) and 2% (*n* = 2), respectively (Figures 2 and 3). The results of sensitivity and specificity of Multiplex PCR for the three studied bacteria indicated general specificity and sensitivity of 72% and 80%, respectively. Bacterial susceptibility and specificity are listed in Table 3.

**TABLE 3:** Specificity and sensitivity of Multiplex PCR

	Specificity	Sensitivity
***S. aureus***	%57.9	**%84.5**
***S. typhi***	%75.8	**%61.6**
***L. monocytogenes***	%100	**%100**
**Total**	%72	**%80**

#### 3.2.1 Antibiotic resistance pattern in *S. aureus*

The results of antibiotic resistance pattern in isolated *S. aureus* strains indicated that all samples were MSSA and that all were sensitive to cefoxitin, gentamicin and tetracycline but the resistance was observed to ampicillin. In the present study, 100 samples of raw milk, pasteurised milk and cheese were studied for the presence of *S. aureus, S. typhi* and *L. monocytogenes* based on culture, biochemical characteristics and amplification of specific genes. Transmission of bacterial species among humans, livestock, farmers and farm workers is increasing dramatically in Europe and many parts of the world. This is of great importance, especially with increasing use of antibiotics, which has increased the resistance to various drugs significantly and has made control and treatment in humans and animals more difficult. Therefore, rising antibiotic resistance is a concern and should be controlled (Patel et al., 2018). Milk is a good environment for *S. aureus* to grow and produce enterotoxin, especially because enterotoxin retains its biological activity even after pasteurisation. *Salmonella* spp is one of the most important infectious agents in humans and animals. The crucial role of infected animal populations as a source for preserving and transmitting Salmonella to humans, especially through contaminated food, is quite clear today (Knight‐Jones et al., 2016). Fecal entry into milk can also be a main cause of milk contamination with Salmonella; this bacterium can enter raw milk from the outer surface of udder and from equipment, environment, bed and water*. L. monocytogenes* has been the focus of many researchers due to its high prevalence and mortality rate (Dhanashekar et al., 2012 Sep 1). This bacterium is also responsible for many food poisoning epidemics, especially in industrialised countries. Recently, a stronger association between listeriosis and dairy consumption has been reported compared to other food products, and pasteurised and unpasteurised milk as well as cheese have been identified as a common source of epidemics. *L.monocytogenes* is able to grow in unpasteurised milk and there is a possibility of increasing number of organisms during storage in milk, storage tanks in farms and silos (Hunt et al., 2012). During the first study conducted in Iran by Jalali and Abedi (2008) regarding *L. monocytogenes* on various foods, the contamination rate of dairy products was 1.3%, which was consistent with our results (2%). In a study by Rodríguez‐Lázaro et al. (2017) in Brazil, *S. aureus* was isolated from 15% of cases that was different from our findings. Van Kessel et al. (2004) conducted a study in 2004 on samples taken from milk containers based on the presence or absence of Salmonella in raw milk. During the isolation of Salmonella by culture technique, the initial results showed that 2.6% of samples were infected with Salmonella. In their study, 20 out of 861 samples of cultured milk were infected with Salmonella, which were viable after enrichment and on the culture medium. These bacteria showed growth, but only two positive samples were obtained from direct culture of bacteria on selective and specific media such as XLD, which indicates the high importance of using pre‐enrichment and enrichment steps. In general, the present study was conducted due to the significance of food pathogens in raw milk, pasteurised milk and cheese since in some areas, dairy consumption is mostly in the form of raw milk and pasteurisation is done at home by individuals or dairy shops that are responsible for sales. It is necessary to assess these milks for the safety of their microbial quality. On the other hand, although there is some degree of monitoring in pasteurised milk, some substances that do not match the microbial quality of milk have been found in them. In this study, the prevalence of *S. aureus* in raw milk was reported to be 10% by culture method and 60% by real‐time qPCR. The results of culture and real‐time qPCR were different, which indicates the need to examine suspicious samples using molecular methods. On the other hand, Salmonella and Listeria are also considered as indicators of raw milk contamination. The specific genes of these two bacteria were reported to be 2% for *L. monocytogenes* and 53% for *S. typhi*, indicating the importance of molecular methods in the diagnosis of such microbial infections. The results of Multiplex PCR showed that 35% (*n* = 35) of bacteria were *S. typhi* and 78% (*n* = 78) were *S. aureus*. Multiplex PCR cannot be used to identify milk‐borne microbes that showed a difference result from real‐time qPCR. A review of past studies in other areas shows that the tanks carrying raw milk do not have the desired hygienic quality. Consumption of raw milk can reduce safety and health quality, which endangers people's health. According to the results of this study, we conclude that *S. typhi, S. aureus* and *L. monocytogenes* can be identified by molecular PCR due to the high sensitivity of the test relative to the culture method. Achieving the result over a short time is one of the advantages of molecular PCR method. However, the possibility of isolating dead bacteria in this method is high and it can be concluded that this approach indicates the presence or absence of bacteria in milk. Therefore, we suggest that the following points:

1. Informing and preventing people from consuming raw milk and products produced from it.
2. Awareness of groups at high risk of infection.
3. This information should be provided to food and medicine authorities and the research should be continued by the university to control contamination
4. Studies should be done on a larger scale.

## ETHICAL APPROVAL STATEMENT

The current study was approved by ethical committee of Ilam University of medical sciences (ID:981017/104).

## CONFLICT OF INTEREST

There is no conflict of interest.

## AUTHOR CONTRIBUTIONS

Mahsa Morovati Bastam: Investigation; methodology; writing‐original draft. Mahsa Jalili: Investigation; project administration. Iraj Pakzad: Formal analysis; software. Abbas Maleki: Data curation; software. Sobhan Ghafourian: Supervision; validation; writing‐review & editing.

### PEER REVIEW

The peer review history for this article is available at https://publons.com/publon/10.1002/vms3.604
